# The Amino-Terminal Domain of GRK5 Inhibits Cardiac Hypertrophy through the Regulation of Calcium-Calmodulin Dependent Transcription Factors

**DOI:** 10.3390/ijms19030861

**Published:** 2018-03-14

**Authors:** Daniela Sorriento, Gaetano Santulli, Michele Ciccarelli, Angela Serena Maione, Maddalena Illario, Bruno Trimarco, Guido Iaccarino

**Affiliations:** 1 Dipartmento di “Scienze Biomediche Avanzate”, Università “Federico II” di Napoli, Via Pansini 5, 80131 Napoli, Italy; danisor@libero.it (D.S.); gsantulli001@gmail.com (G.S.); trimarco@unina.it (B.T.); 2 Department of Medicine, Albert Einstein College of Medicine, Montefiore University Hospital, 1300 Morris Park Avenue, Bronx, NY 10461, USA; 3 Dipartimento di Medicina, Chirurgia e Odontoiatria “Scuola Medica Salernitana”/DIPMED, Università degli Studi di Salerno, Via S. Allende, 84081 Baronissi (SA), Italy; mciccarelli@unisa.it; 4 Dipartmento di “Scienze Mediche Traslazionali”, Università “Federico II” di Napoli, Via Pansini 5, 80131 Napoli, Italy; mercoledi85@gmail.com (A.S.M.); illario@unina.it (M.I.)

**Keywords:** cardiac hypertrophy, transcription factors, calmodulin, GRK

## Abstract

We have recently demonstrated that the amino-terminal domain of G protein coupled receptor kinase (GRK) type 5, (GRK5-NT) inhibits NFκB activity in cardiac cells leading to a significant amelioration of LVH. Since GRK5-NT is known to bind calmodulin, this study aimed to evaluate the functional role of GRK5-NT in the regulation of calcium-calmodulin-dependent transcription factors. We found that the overexpression of GRK5-NT in cardiomyoblasts significantly reduced the activation and the nuclear translocation of NFAT and its cofactor GATA-4 in response to phenylephrine (PE). These results were confirmed in vivo in spontaneously hypertensive rats (SHR), in which intramyocardial adenovirus-mediated gene transfer of GRK5-NT reduced both wall thickness and ventricular mass by modulating NFAT and GATA-4 activity. To further verify in vitro the contribution of calmodulin in linking GRK5-NT to the NFAT/GATA-4 pathway, we examined the effects of a mutant of GRK5 (GRK5-NTPB), which is not able to bind calmodulin. When compared to GRK5-NT, GRK5-NTPB did not modify PE-induced NFAT and GATA-4 activation. In conclusion, this study identifies a double effect of GRK5-NT in the inhibition of LVH that is based on the regulation of multiple transcription factors through means of different mechanisms and proposes the amino-terminal sequence of GRK5 as a useful prototype for therapeutic purposes.

## 1. Introduction

Left ventricular hypertrophy (LVH) is an adaptive response of the heart to stress that eventually, and progressively, turns into maladaptive, evolving towards cardiac dysfunction and heart failure [1,2,3,4]. Available therapies targeting the pro-hypertrophic pathways, including angiotensin converting enzyme (ACE) inhibitors and β-adrenergic receptor (β-AR) blockers, can reduce hypertrophy significantly, but not completely [5]. During the past decade, scientific research has focused on the identification of new targets for the treatment of cardiac hypertrophy [5,6,7]. In particular, alterations in intracellular calcium fluxes activate intracellular pathways that are involved in the progression of LVH and myocardial remodeling [8]. Intracellular Calcium fluxes are the primary component of excitation-contraction coupling and maintain heart contractility [9,10], but can also ignite nuclear gene transcription. In particular, several transcription factors, including nuclear factor of activated T cells (NFAT), are calcium-dependent [11]. Calcium binds calmodulin (CaM) and activates the serine/threonine calcium-calmodulin phosphatase calcineurin. Activated calcineurin, in turn, dephosphorylates the transcription factor NFAT, which quickly moves from cytosol to the nucleus [12,13]. Here NFAT regulates the transcription of genes involved in the development of cardiac hypertrophy, including Atrial Natriuretic Factor, TNF-α, and Endothelin-1 [14]. NFAT works in association with other transcription factors, such as GATA-4. This latter is activated by ERK phosphorylation [15], but also Ca/CaM dependent pathway can enable it.

Previously, we demonstrated that the RH domain of G protein-coupled receptor kinase 5 (GRK5) inhibits NFκB transcriptional activity through binding of the inhibitory protein IκBα [16,17,18]. In vivo, the intra-myocardial injection of an adenovirus encoding for the amino-terminal domain of GRK5 (AdGRK5-NT), which comprises the RH domain, reduces LVH in spontaneously hypertensive rats (SHR) in a blood pressure-independent manner through the inhibition of NFκB transcription activity [19]. Interestingly, the same amino-terminal sequence of GRK5 that contains the RH domain also flanks a calmodulin binding site [20]. Therefore, it is possible to speculate that GRK5-NT is involved in the regulation of calcium-calmodulin dependent events of activation of transcription factors. This study aims to evaluate whether GRK5-NT can regulate the activation of transcription factors NFAT and GATA-4 through the interaction with calcium-calmodulin dependent signaling pathways.

## 2. Results

### 2.1. GRK5-NT Regulates the Activation of Calcium-Calmodulin Dependent Transcription Factors In Vitro

**Figure 1:**
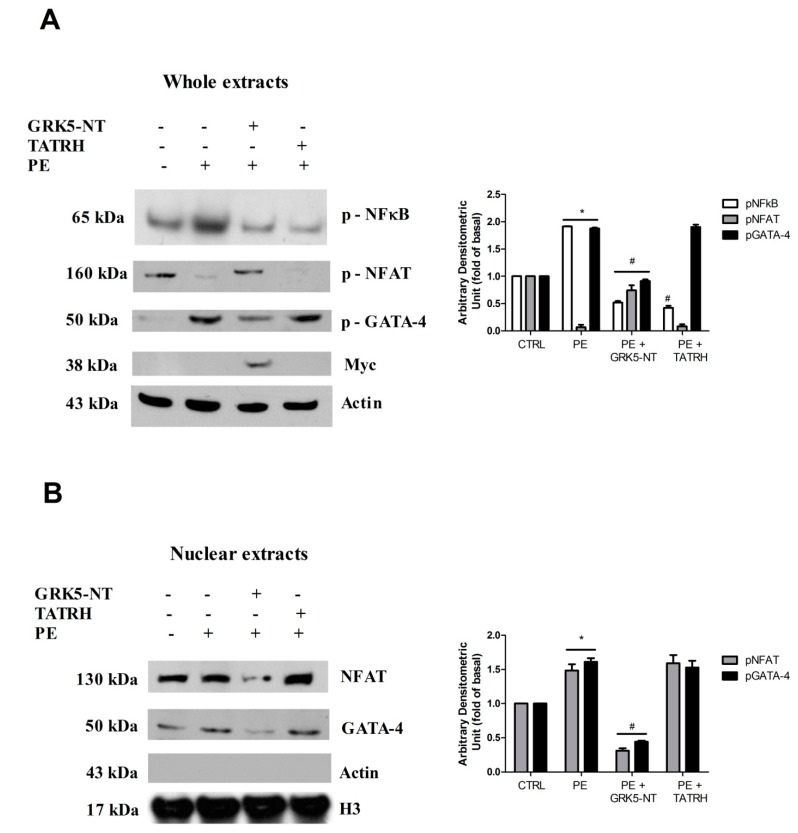
The amino-terminal domain of the G Protein Coupled Receptor Kinase 5 (GRK5-NT) regulates the activation of calcium-calmodulin dependent transcription factors in vitro. (**A**) In cultured cardiomyoblasts H9C2, hypertrophy was induced by chronic stimulation with Phenylephrine (PE) and the activation of NFκB, nuclear factor of activated T cells (NFAT), and GATA-4 in response to PE was evaluated by western blot. PE triggered the phosphorylation of GATA-4, NFκB and the dephosphorylation of NFAT. The overexpression of GRK5-NT reduces such phenomenon while TAT-RH, which has only the RH domain of GRK5, did not modify PE-induced GATA-4 and NFAT activation albeit being effective on NFκB activation. Images are representative of three independent experiments. Densitometric analysis is shown in bar graph as mean ± SD; * *p* < 0.05 vs. Control and ^#^ *p* < 0.05 vs. PE; (**B**) nuclear accumulation of NFAT and GATA-4 was evaluated by western blot in nuclear extracts from H9C2 cells. GRK5-NT reduced the PE-dependent nuclear translocation of these factors while TATRH is not able to exert the same effect. Actin was used as control of nuclear extracts purity. Images are representative of three independent experiments. Densitometric analysis is shown in bar graph as mean ± SD; * *p* < 0.05 vs. Control and ^#^ *p* < 0.05 vs. PE.

In cultured cardiomyoblasts, hypertrophy was induced by chronic PE stimulation. PE induced the activation of the transcription factors GATA-4, NFκB, and NFAT (Figure 1A). The overexpression of GRK5-NT inhibited the activation of these transcription factors in response to PE (Figure 1A). On the contrary, TAT-RH, which is a peptide that reproduces only the RH domain of GRK5 lacking the amino-terminal domain, did not affect GATA-4 and NFAT activation in response to PE, but was active on NFκB inhibition, as consistent with our previous findings [18]. These data suggest that GRK5-NT is able to regulate calcium-calmodulin-dependent transcription factors, through means of its amino-terminal domain and independently from the RH domain. To verify these findings, we assessed NFAT and GATA 4 nuclear translocation. Strikingly, GRK5-NT overexpression reduced their nuclear accumulation in response to PE, whereas TATRH had no effects (Figure 1B).

### 2.2. GRK5-NT Inhibits NFAT Activation by Competing for Binding to Calmodulin

**Figure 2:**
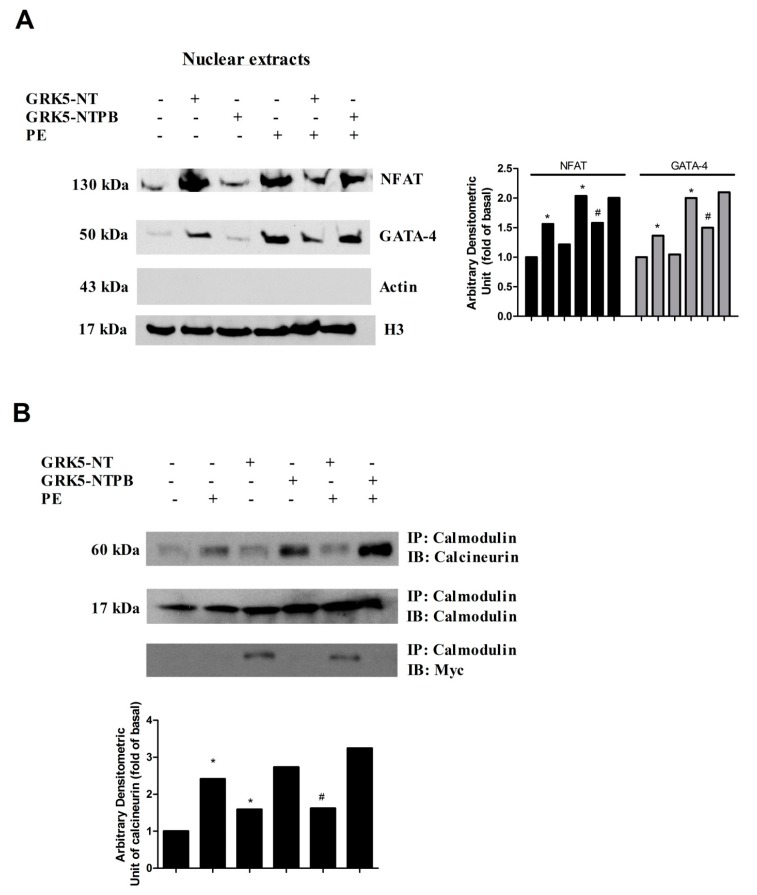
The amino-terminal domain of the G Protein Coupled Receptor Kinase 5 (GRK5-NT) inhibits NFAT activation by sequestrating calmodulin. (**A**) Cardiomyoblasts were transfected with a plasmid encoding GRK5-NT or GRK5-NTPB and NFAT and GATA-4 nuclear translocation was evaluated by western blot in presence and absence of phenylephrine (PE). GRK5-NTPB had no significant effect on nuclear translocation and activation of NFAT and GATA4; instead GRK5-NT inhibited such phenomenon. Actin was used as control of nuclear extracts purity and histone 3 (H3) was used as loading control. Densitometric analysis is shown in bar graph as mean ± SD, * *p* < 0.05 vs. Control and ^#^ *p* < 0.05 vs. PE; (**B**) cardiomyoblasts were transfected with a plasmid encoding GRK5-NT or GRK5-NTPB and calmodulin was precipitated in whole lysates from these cells. Calcineurin was evaluated by western blot. GRK5-NT reduced calmodulin/calcineurin interaction. GRK5-NTPB had no effect on such interaction. All images are representative of three independent experiments. Densitometric analysis is shown in bar graph as mean ± SD, * *p* < 0.05 vs. Control and ^#^ *p* < 0.05 vs. PE.

To further clarify in vitro the molecular mechanisms by which GRK5-NT regulates the activation of calcium-calmodulin-dependent transcription factors, we tested the hypothesis that GRK5-NT regulates these factors by sequestrating calmodulin. To this aim, cardiomyoblasts were transfected with a plasmid encoding a mutated form of GRK5 in the calmodulin binding site (GRK5-NTPB), which enables the kinase to bind calmodulin [21]. Figure 2A shows that, when compared to GRK5-NT, GRK5-NTPB had no significant effect on PE-induced nuclear translocation of GATA-4 and NFAT, strongly suggesting that GRK5-NT regulates GATA-4 and NFAT activation by competing with calcineurin for binding to calmodulin. Therefore, we evaluated the effects of GRK5-NT and GRK5-NTPB on calmodulin/calcineurin interaction via immunoprecipitation and western blot. We observed that PE induces such interaction, which is not affected by GRK5-NTPB, but is markedly reduced by GRK5-NT (Figure 2B).

### 2.3. GRK5-NT Regulates the Activation of Calcium-Calmodulin Dependent Transcription Factors In Vivo

**Figure 3:**
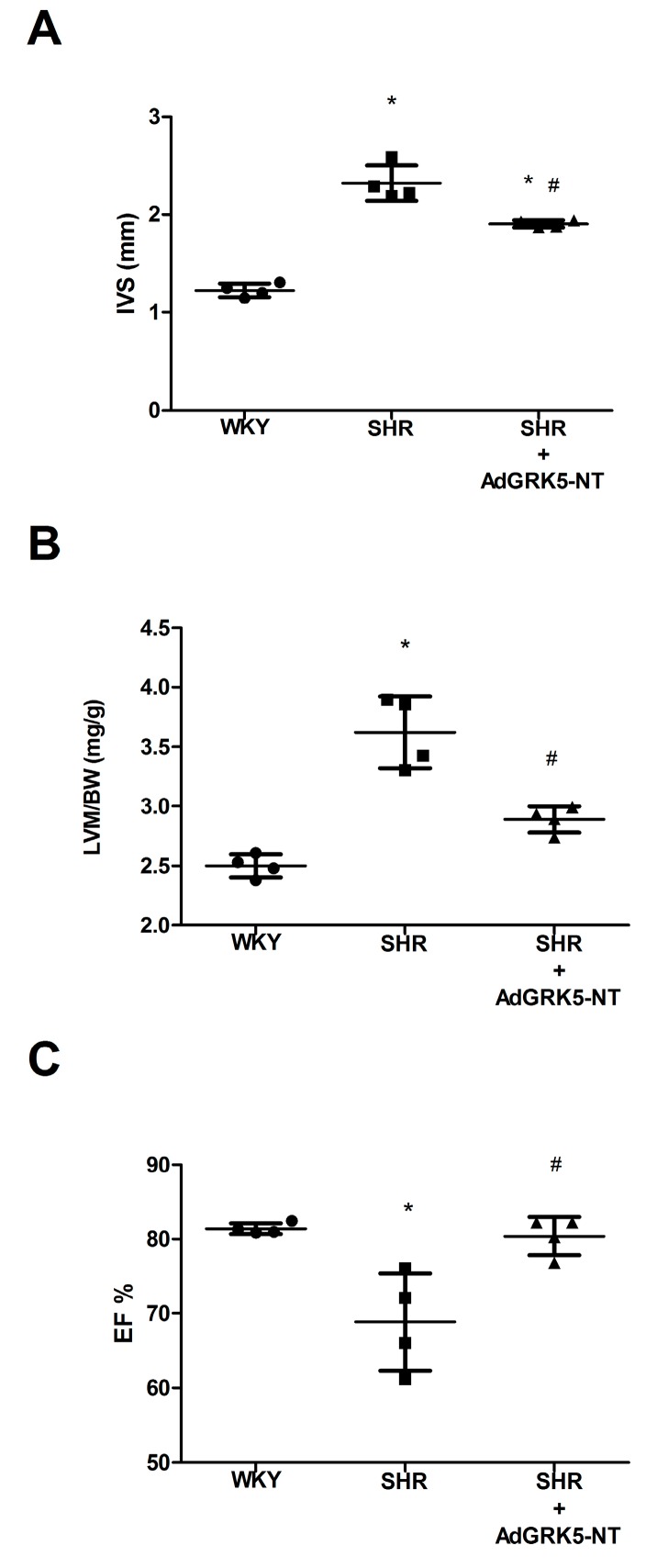
The amino-terminal domain of the G Protein Coupled Receptor Kinase 5 (GRK5-NT) inhibits calcium-calmodulin dependent transcription factors in vivo. (**A**–**C**) Spontaneously hypertensive and hypertrophic rats (SHR) were treated with an intra-myocardial injection of an adenovirus encoding for GRK5-NT (AdGRK5-NT) or Lac-Z (AdLac-Z), as described in methods, and cardiac hypertrophy was evaluated by echocardiography. 21 days after injection, a reduction of IVS (**A**) and LVM/BW (**B**), was found in AdGRK5-NT versus SHR controls. Cardiac function was recovered in treated SHR vs. SHR (**C**) * *p* < 0.05 vs. WKY and ^#^ *p* < 0.05 vs. SHR + AdLac-Z.

**Figure 4:**
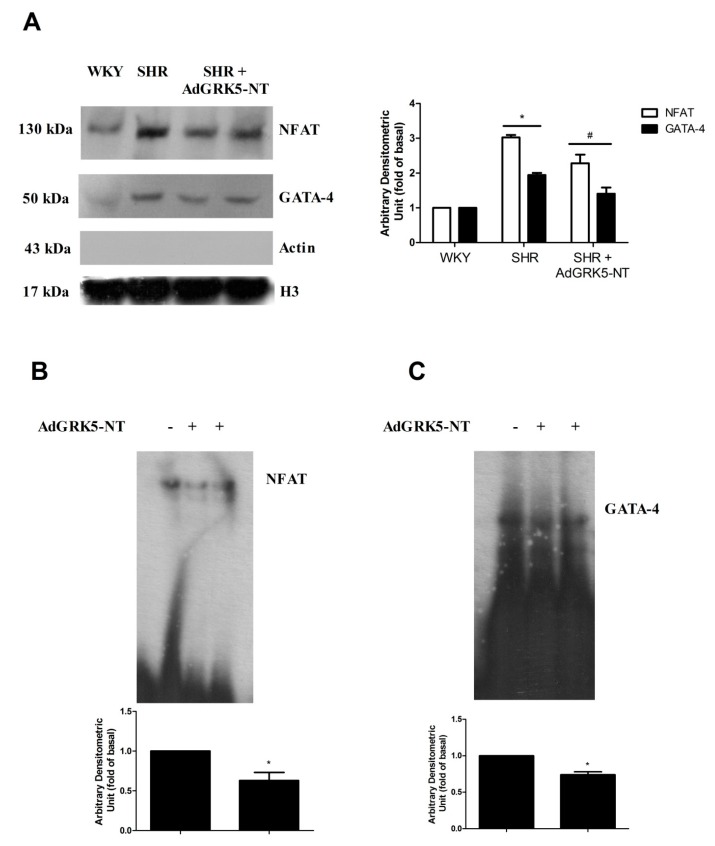
The amino-terminal domain of the G Protein Coupled Receptor Kinase 5 (GRK5-NT) regulates NFAT and GATA-4 activity in vivo. (**A**) The nuclear translocation of NFAT and GATA-4 was evaluated by immunoblot in rat hearts. In hypertrophic SHR rats, there was a significant accumulation of NFAT and GATA-4 in nuclear extracts respect to WKY. The treatment with AdGRK5-NT significantly reduced nuclear translocation of these factors. Densitometric analysis is shown in bar graph as mean ± SD. Actin was used as control of nuclear extracts purity; * *p* < 0.05 vs. WKY and ^#^ *p* < 0.05 vs. SHR + AdLac-Z; (**B**,**C**) to confirm the inhibition of transcription factors activity, we analyzed NFAT and GATA-4 ability to bind DNA by EMSA in control and treated SHR. GRK5-NT reduced both NFAT (**B**) and GATA-4 (**C**) activity in response to hypertrophy. All of the images are representative of at least three independent experiments. Densitometric analysis is shown in bar graph as mean ± SD; * *p* < 0.05 vs. SHR + AdLac-Z.

To confirm our in vitro data, we evaluated the effects of GRK5-NT on NFAT and GATA-4 activation in an animal model of hypertrophy. Spontaneously hypertensive rats (SHR) underwent intra-myocardial injections of an adenovirus encoding for GRK5-NT (AdGRK5-NT) or Lac-Z as control (AdLac-Z), as previously described [19]. Rats were monitored for three weeks by CUS to assess the effect of such treatment on LVH. After 21 days, LVH was significantly reduced, as underlined by the reduction of IVS (Figure 3A) and LVM/BW (Figure 3B). Moreover, cardiac function was recovered in treated rats (Figure 3C). Hearts were then collected and the nuclear translocation of NFAT and GATA-4 was assessed by immunoblot. In hypertrophic SHR rats, there was a significant accumulation of NFAT and GATA-4 in the nucleus as compared with WKY (Figure 4A). The treatment with AdGRK5-NT significantly inhibited such nuclear translocation (Figure 4A) thereby indicating that GRK5-NT can reduce NFAT and GATA-4 activation in response to hypertrophy in vivo. Importantly, this finding was confirmed by EMSA assay, which shows that GRK5-NT significantly reduces both NFAT (Figure 4B) and GATA-4 (Figure 4C) ability to bind DNA in an established model of cardiac hypertrophy (i.e., SHR).

## 3. Discussion

**Figure 5:**
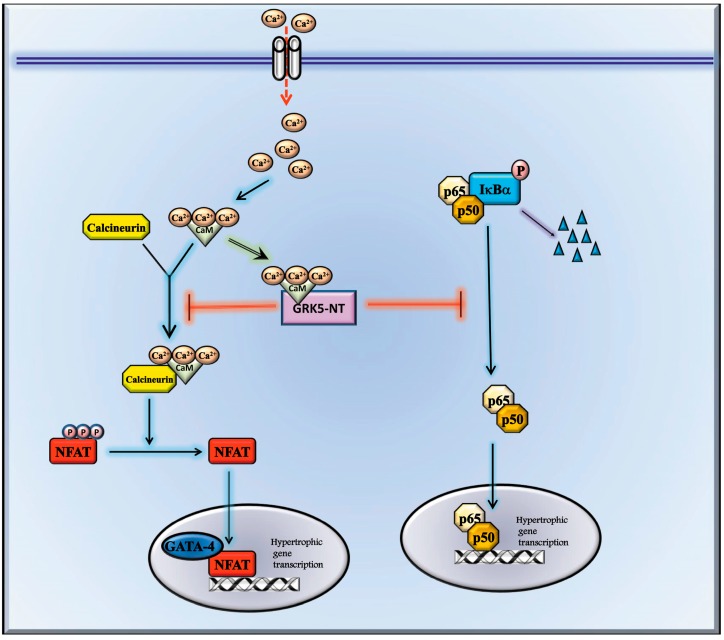
Schematic representation of the molecular mechanism. GRK5-NT regulates GATA-4 and NFAT activation by competing with calcineurin for binding to calcium-calmodulin. PE induces an increase of intracellular calcium levels. Calcium binds calmodulin and activates calcium-calmodulin dependent factors such as calcineurin. Calcineurin, in turn, dephosphorylates NFAT leading to NFAT nuclear translocation and activation of gene transcription. GRK5-NT binds calcium-calmodulin, through the calmodulin binding site (CBS), thus inhibiting its binding to calcineurin. Inactivated calcineurin is not anymore able to dephosphorylate NFAT and this causes the inhibition of NFAT dependent gene transcription and the inhibition of NFAT dependent GATA-4 activation. Besides this effect of GRK5-NT, the peptide is also able to inhibit IκB degradation, thus preventing NFκB transcription activity.

Several transcription factors are involved in the regulation and development of LVH [11,22,23] by regulating the expression of critical hypertrophic genes in response to specific stimuli [24,25]. We have recently demonstrated that the treatment with GRK5-NT inhibits NFκB activity in hypertrophied hearts [19]. Here, we describe a novel level of inhibition of LVH by using the ability of the GRK5 sequence to regulate CaM signaling and to prevent NFAT and GATA-4 activation. Indeed, we show that GRK5-NT regulates the activation of the calcium-calmodulin-dependent transcription factor, NFAT, and its cofactor GATA-4, through binding to calmodulin (Figure 5). Other calcium-calmodulin dependent pathways are involved in the development of cardiac hypertrophy, such as CaMKs signaling, and we cannot exclude the possibility that they could be affected by GRK5-NT. However, in this study, we focused on NFAT and its co-factor GATA-4, since they are among the main cardiac transcription factors whose activation is strictly dependent on calcium-calmodulin interactions. Hence, our data indicate that within the GRK5-NT sequence, there are two regions with the potentiality to regulate cardiac hypertrophy in two different ways: by inhibiting NFκB through the binding of the RH domain to IκBα [19] and by inhibiting NFAT through the binding and sequestration of calmodulin in the amino-terminal domain. GRK5-NT can simultaneously affect these intracellular signaling pathways since our data show that it can inhibit both NFκB and NFAT when compared with TATRH.

A direct association between NFAT and NFκB activity in cardiac myocytes has been shown to promote cardiac hypertrophy and ventricular remodeling [26]. In particular, since the inhibition of NFκB with IκBαM or the dominant negative of IKKβ reduces NFAT activity both in vitro and in vivo [26], it is likely that the inhibitory effect of GRK5-NT on NFAT activity could be a consequence of GRK5-NT dependent inhibition of NFκB. Actually, in our model, we did not find a mechanistic association between NFκB and NFAT activity. Indeed, while GRK5-NT regulates both NFκB and NFAT activity, TAT-RH—which specifically inhibits NFκB signaling—does not modify NFAT or GATA-4 activation. Such discrepancy could be attributable to the different ways of inhibition of NFκB transcriptional activity. Indeed, the inhibition of NFκB that was obtained via overexpressing IκBαM or a dominant negative IKKβ is mainly based on the inhibition of IκB phosphorylation. In our model, the effects of GRK5-NT are instead based on a protein-protein interaction without interfering with IκB phosphorylation [16]. Such binding leads to the generation of a macromolecular complex that could prevent the binding of NFAT. Furthermore, GRK5-NT regulates NFAT in different manner respect to NFκB that is based on the sequestration of calmodulin through the amino-terminal domain. These findings suggest that GRK5-NT dependent inhibition of NFAT is not due to NFκB regulation, but the peptide is able to regulate both intracellular signalings simultaneously.

Here, we show that GRK5-NT also regulates GATA-4, which is not itself a calcium-calmodulin dependent transcription factor. It has been shown that its activation induced by PE stimulation is coupled with serine phosphorylation by Extracellular signal–regulated kinase 2 (ERK2) [15]. However, besides phosphorylation, the transcriptional activity of GATA4 is also regulated through interaction with other cofactors such as p300, MEF2, SRF, and NFAT [11,24]. Among them, the interaction with NFAT is noteworthy since NFAT plays a critical role in activating the hypertrophic gene program, and this activity is partly dependent on its interaction with GATA4 [24]. These findings suggest the functional importance of calcium signaling also in the activation of GATA4 and support our finding on GRK5-NT dependent GATA-4 inhibition through the modulation of calcium-calmodulin signaling.

Previous reports show that GRK5 also induces cardiac hypertrophy [27,28] by activating NFAT-dependent gene transcription [29]. In this context, GRK5-NT exerts opposite effects compared with the full-length sequence of GRK5. This is because GRK5-NT includes the amino-terminal domain of GRK5 (first 170 aminoacidic sequence) and lacks the catalytic domain that is instead responsible for GRK5 effects on gene transcription. Indeed, GRK5 exacerbates cardiac hypertrophy by phosphorylation of different substrates (plasma membrane receptors and transcription factors) [27,28,29,30]. On the contrary, GRK5-NT acts by protein-protein interaction within the RH domain and calmodulin binding within the amino-terminal sequence. However, we cannot exclude the possibility that GRK5-NT could interfere with endogenous GRK5 signaling. Literature is quite discordant on the effects of calmodulin binding on GRK5 signaling. Indeed, some reports show that CaM binding to GRK5 inhibits its catalytic activity [20], while others suggest that this interaction inhibits the binding of GRK5 to plasma membrane favoring its nuclear translocation to induce HDAC phosphorylation [27]. Pitcher et al. which generate the mutant plasmid of GRK5 (GRK5-NTPB) showed that the binding of nuclear Calcium-Calmodulin to the amino-terminal CaM-binding site of GRK5 is required for nuclear export [21]. Thus, further studies are needed to better clarify this issue. Our data show that GRK5-NT in basal condition exerts a pro-hypertrophic effect inducing NFAT nuclear translocation, and this appears to be in contrast with the effect of the peptide in response to PE. Hypertrophic stimuli increase the intracellular calcium levels and enhance the affinity of calmodulin to its substrates. Thus the effect of GRK5-NT to compete with calcineurin for calmodulin binding could take place mainly in response to stimuli. In resting conditions, GRK5-NT may interfere with other physiological pathways and indirectly regulate NFAT nuclear translocation. Taken together, our data demonstrate the ability of GRK5-NT to control several fundamental cardiac transcription factors in response to hypertrophy by different mechanisms of action which involve different domains of GRK5. Specifically, here we demonstrate the ability of GRK5-NT to regulate calcium-calmodulin dependent transcription factors, proposing GRK5 as a potential regulator of calcium signaling through its calmodulin binding site. In conclusion, our data confirm the usefulness of GRK5-NT for the treatment of LVH, suggesting the GRK5-NT sequence as a prototype for the generation of small molecules that are to be used for therapeutic applications.

## 4. Materials and Methods

### 4.1. Cell Culture

A cell line of cardiac myoblasts (H9C2) was maintained in culture in Dulbecco Modified Eagle Medium (DMEM) supplemented with 10% FBS at 37 °C in 95% air-5% CO_2_.

### 4.2. Plasmids

p-GRK5-NT is a pcDNA3.1 myc/his plasmid encoding the amino-terminal domain of GRK5, including the RH domain (aa 1-176), and was described previously [16]; p-GRK5-NTPB, which is a plasmid encoding a mutant form of GRK5 which lacks the ability to bind calmodulin, was a kind gift of Julie Pitcher (University College London, London, UK) and was used as template for cloning p-GRK5-NT mutant (GRK5-NT mut), as previously described [16]; TAT-RH was described previously [18]. All experiments were performed using TAT alone and empty pcDNA3.1 as controls. Transient transfection of the plasmids was performed, as previously described [31], using Lipofectamine 2000 from Invitrogen (Thermo Fisher Scientific, Waltham, MA, USA) in 70% confluent H9C2, accordingly to manufacturer instructions.

### 4.3. Western Blot

The experiments were performed as described previously [16,32]. H9C2 were treated with 10^−7^ M phenylephrine (PE, Sigma-Aldrich Corporation, St. Louis, MO, USA) for 24 h. In some experiments, cells were treated with the synthetic peptide TAT-RH, which only reproduce the RH domain of GRK5, as described previously [18] or transfected with plasmids encoding for GRK5-NT or its mutant in the amino-terminal calmodulin binding. At the end of the treatment, cells were lysed in RIPA/SDS buffer, and protein concentration was determined by using Pierce BCA assay kit (Thermo Fisher Scientific, Waltham, MA, USA) [33,34]. Total extracts were electrophoresed by SDS/PAGE and transferred to nitrocellulose [35]. The antibodies anti-NFATc4 (B-2) (SC-271597), GATA-4 (G-4) (sc-25310), p-NFATc4 (80.S168/170) (sc-135770), p-GATA-4 (H-4) (sc-377543), β-actin (C-4) (sc-47778), and histone H3 (FL-136) (sc-10809) were from Santa Cruz Biotechnology (Santa Cruz Biotechnology, Inc, Dallas, TX, USA). In some experiments, nuclear proteins were isolated from heart samples as previously described [16]. Densitometric analysis was performed using Image Quant 5.2 software (Molecular Dynamics Inc., Caesarea, Israel). Images are representative of at least three independent experiments quantified and corrected for appropriate loading control.

### 4.4. In Vivo Study

Experiments were carried out accordingly with the Federico II University Ethical Committee on 12-week–old spontaneously hypertensive male rats SHR (AdLac-Z *n* = 6, and AdGRK5-NT *n* = 6) and 4 normotensive Wistar-Kyoto (WKY) rats as control. The animals were obtained from Charles River (Wilmington, MA, USA) and had access to water and food ad libitum. Anesthesia was obtained through isoflurane (4%). After the induction of anesthesia, rats were orotracheally intubated, the inhaled concentration of isoflurane was reduced to 1.8%, and the lungs were mechanically ventilated (New England Medical Instruments Scientific, Inc., Chelmsford, MA, USA). The chest was opened under sterile conditions through a right parasternal mini-thoracotomy to expose the heart. Then, we performed four injections (50 μL each) of AdGRK5-NT (10^10^ pfu/mL) or AdLac-Z (10^10^ pfu/mL) as control, into the cardiac wall (anterior, lateral, posterior, and apical), as previously validated [19]. Finally, the chest wall was quickly closed in layers, and animals were observed and monitored until recovery.

### 4.5. Cardiac Ultrasounds (CUS)

Transthoracic CUS was performed at days 0, 7, 14, and 21 after surgery using a dedicated small-animal high-resolution imaging system (VeVo 770, Visualsonics, Inc., Amsterdam, The Netherlands). The rats were anesthetized with isoflurane (4%) inhalation and maintained by mask ventilation, as described above. The chest was shaved with a depilatory cream (Veet, Reckitt-Benckiser, Milan, Italy). Left ventricular (LV) end-diastolic and LV end-systolic diameters (LVEDD and LVESD, respectively) were measured at the level of the papillary muscles from the parasternal short-axis view [36,37]. Intraventricular septal (IVS) and LV posterior wall thickness (PW) were measured at the end of the diastolic phase. LV mass (LVM) was obtained, as described and corrected by body weight [19,36]. All of the measurements were averaged on at least five consecutive cardiac cycles and were analyzed by investigators that were blinded to treatment.

### 4.6. EMSA

Electrophoretic mobility shift assay (EMSA) was performed on lysates from treated hearts as previously described [16].

### 4.7. Statistical Analysis

All data are presented as mean ± SEM. Two-way ANOVA with Bonferroni post hoc test was performed to compare the different parameters between groups. A *p* value < 0.05 was considered significant. Statistical analysis was performed using GraphPad Prism version 5.01 (GraphPad Software, Inc., San Diego, CA, USA).

## 5. Conclusions

This study identifies a double effect of GRK5-NT in the inhibition of LVH that is based on the regulation of multiple transcription factors through means of different mechanisms. Indeed, GRK5-NT is able to inhibit NFκB activity through the interaction of RH domain with IkBα and to inhibit NFAT activity by means of calcium-calmodulin sequestration. In conclusion, here we propose the amino-terminal sequence of GRK5 as a useful prototype for therapeutic purposes.
